# Mushroom consumption and hyperuricemia: results from the National Institute for Longevity Sciences-Longitudinal Study of Aging and the National Health and Nutrition Examination Survey (2007-2018)

**DOI:** 10.1186/s12937-023-00887-0

**Published:** 2023-11-22

**Authors:** Djibril M. Ba, Shu Zhang, Yukiko Nishita, Chikako Tange, Tian Qiu, Xiang Gao, Joshua Muscat, Rei Otsuka

**Affiliations:** 1 Department Public Health Sciences, Penn State College Medicine, Hershey, USA; 2 Department of Epidemiology of Aging, Research Institute, National Center for Geriatrics and Gerontology, Obu, Japan; 3 Department of Nutrition and Food Hygiene, School of Public Health, Institute of Nutrition, Fudan University Shanghai, Shanghai, China

**Keywords:** NHANES, NILS-LSA, Mushrooms, Hyperuricemia, U.S., Japan

## Abstract

**Background:**

Prior study reported that mushroom consumption was associated with a lower incidence of hyperuricemia, but there is limited evidence on this association. We conducted a collaborative study to investigate the association between mushroom intake and hyperuricemia in middle-aged and older populations.

**Methods:**

We used data from the National Health and Nutrition Examination Survey (NHANES) in the U.S. (2007–2018) and the National Institute for Longevity Sciences-Longitudinal Study of Aging (NILS-LSA) in Japan (1997–2012). Consumption of mushroom (g/day) were measured by one- or two-day dietary recall in NHANES and by 3-day dietary records in the NILS-LSA. Hyperuricemia was defined using uric acid levels as > 420 μmol/L and > 350 μmol/L in NHANES for men and women, respectively; in the NILS-LSA, serum uric acid was repeatedly measured at baseline and follow-up surveys. Hyperuricemia was defined as uric acid levels > 416.4 μmol/L for men and ≥ 356.9 μmol/L for women. Logistic regression models in NHANES (cross-sectionally) and Generalized Estimation Equations in NILS-LSA (longitudinally) were performed.

**Results:**

A total of 5,778 NHANES participants (mean (SD) age: 53.2 (9.6) years) and 1,738 NILS-LSA (mean (SD) age: 53.5 (11.2) years) were included. Mushrooms were consumed by 5.7% of participants in NHANES and 81.2% in NILS-LSA. We did not observe a significant association between mushroom intakes and hyperuricemia in the NHANES men and women. However, in the NILS-LSA, compared to non-consumers, a higher mushroom intake was associated with a lower risk of incident hyperuricemia in men under 65 years old. The adjusted odds ratio (95% CI) for non-consumers, participants with middle, and the highest consumption of mushrooms were 1.00 (Ref.), 0.77 (0.44, 1.36), and 0.55 (0.31, 0.99), respectively (*P*-trend = 0.036). No association was found in women in NILS-LSA.

**Conclusions:**

Mushroom consumption was associated with a lower risk of incident hyperuricemia in Japanese men.

**Supplementary Information:**

The online version contains supplementary material available at 10.1186/s12937-023-00887-0.

## Introduction

Hyperuricemia, as an indicator for cardiovascular diseases (especially hypertension) [1–3], may occur when there is an elevated uric acid (UA) level in the blood [2]. Causes of elevated uric acid levels include unhealthy diets, such as high purine diets (high consumption of red meat and processed meats [4]). Conversely, a high consumption of plant-based foods such as vegetables and fruits, which are low in purines, may help to lower UA levels [5].

Growing evidence regarding the potential health benefits of mushrooms as disease-preventing agents, such as anti-neoplastic and anti-neurodegenerative benefits, and treatment agents, has gained special research attention during the last decades [6–9]. For centuries, they have been widely consumed as medicinal products in Asian countries such as Japan, China, and Korea [9]. Edible mushrooms have become more attractive in both Western and non-Western countries as functional foods because of their unique nutritional value and their role in a healthful diet [10]. Mushrooms are rich sources of many important nutrients and bioactive compounds. They are low in energy, high in fiber, and contain essential vitamins (e.g., riboflavin, niacin, vitamin D) [11–13], polysaccharides like β-glucans, potential prebiotics, and gut microbiota modulators [14], as well as minerals including selenium, copper, and potassium [15, 16]. Of particular importance, they are also a rich source of specific potent antioxidants, including ergothioneine (Ergo), which cannot be synthesized by humans [17, 18] and glutathione. Ergo is a unique antioxidant with cytoprotective and anti-inflammatory properties [9], playing a significant role in the prevention of oxidative stress, thus reducing the risk of chronic diseases and premature deaths [19].

A previous study using an animal model demonstrated that intervention with xanthine inhibitors extracted from oyster mushroom fruiting bodies reduced serum urate levels [20]. Additionally, a recent cohort study conducted in China showed that mushroom consumption was associated with a lower risk of incident hyperuricemia [21]. To the best of our knowledge, apart from the previously mentioned Chinese study, there are no other prospective studies with long-term follow-up based on community-based populations that have explored the association between mushroom consumption and the risk of hyperuricemia. Furthermore, the association needs to be explored in populations of different ethnicities, considering differences in diet and lifestyle habits. Therefore, the aim of the present study was to investigate the associations between mushroom consumption and hyperuricemia in middle-aged and older Japanese and American community-dwellers, using data from the National Institute for Longevity Sciences-Longitudinal Study of Aging (NILS-LSA) in Japan (with a 15-year follow-up) and 2007–2018 data from the National Health and Nutrition Examination Survey (NHANES), a nationally representative cross-sectional survey in the U.S.

## Methods

### Data source and study population

The NHANES is a continuing series of cross-sectional surveys, a nationally representative health survey conducted by the National Center for Health Statistics (NCHS) of the Centers for Disease Control and Prevention. The NHANES is a program of studies designed to examine the health and nutritional status of adults and children in the U.S. [22]. The surveys use a multistage, probability sampling design to develop a population sample that is representative of the 50 states of the U.S., including the District of Columbia on the basis of key demographic factors. The program is designed to examine adults' and children's health and nutritional status of a representative sample of the civilian, non-institutionalized U.S. population [22]. Beginning 1999, the survey became a continuous program that has a developing focus on a variety of health and nutrition measurements to accommodate emerging needs [22]. It is a national representative sample of the civilian, non-institutionalized U.S. population located in counties across the U.S. [22]. In other words, the survey does not include persons residing in nursing homes, institutionalized persons, or U.S. citizens living abroad. The surveys include demographic, socioeconomic status, dietary, and health-related questions. The NHANES program also includes physical examination component performed at the mobile examination center (MEC) and a series of laboratory tests. Details on the NHANES Laboratory/Medical Technologists Procedures and Anthropometry Procedures are described elsewhere [23]. The survey protocol is approved annually by the NCHS Research Ethics Review Board, and all participants provided written informed consent [24]. Details information about the dietary interview portion has been published previously [25].

Since NHANES data are de-identified and publicly available, thus, the Institutional Review Board at the researchers’ institution does not consider this to be human subject research. Therefore, human subjects’ approval was not necessary. Of 23,226 participants of NHANES aged ≥ 40 from 2007–2018, 609 participants with implausible daily energy intake (< 800 kcal or > 4,200 kcal for men and < 500 kcal or > 3,500 kcal for women), 2,257 participants with missing uric acid levels, 1,624 with incomplete dietary data, 1,258 with history of gout or missing, 8,091 with history of heart disease, stroke, drugs for gout, antihypertensive, anti-diabetic, 210 with impaired kidney function (estimated glomerular filtration rate, eGFR, < 60 mL/min/1.73m^2^), and 3,399 with missing covariates of interest were excluded. Thus, 5,778 American individuals (2,934 men and 2,844 women) were analyzed in this study.

The NILS-LSA is a Japanese population-based prospective cohort study designed to investigate the processes of normal aging and the development of age-related diseases. The study recruited participants using age- and sex-stratified random sampling from Obu and Higashiura Town in Aichi Prefecture, Japan. The first-wave examination of the NILS-LSA was conducted from November 1997 to April 2000, enrolling 2,267 individuals, all aged between 40 and 79 years. Subsequent to this initial assessment, participants underwent biennial follow-up evaluations, and participants (aged 40–79 years) who were unable to continue their participation were replaced by new, randomly recruited, age- and sex-matched individuals. Notably, individuals aged 40 years were annually recruited. Details of the NILS-LSA have been reported previously [26]. This research involving human participants strictly adhered to the principles outlined in the Declaration of Helsinki. The Committee on the Ethics of Human Research of the National Center for Geriatrics and Gerontology approved the study protocol (No. 1665–2), and written informed consent was obtained from all participants. All participants provided written informed consent for data collection and analysis before participating in the study.

The participants in this study were selected from the first to the seventh waves of the NILS-LSA, spanning from November 1997 to July 2012. In this study, the initial participation of each participant was used as the baseline, and the follow-up began from the date of their baseline participation. Among the initial 3,983 participants, 3,849 individuals with complete dietary survey data were included, while 1,363 participants who took part in less than two follow-up surveys were excluded. Additionally, 508 individuals with a history of kidney disease, gout, hyperuricemia, or impaired kidney function (eGFR, < 60 mL/min/1.73m^2^) at baseline, and 180 individuals with unavailable self-reported history of stroke, heart disease, and cancer data at baseline, or a history of any of these diseases, were also excluded. Furthermore, 19 participants had incomplete baseline covariate data, and 41 were taking medications (such as thiazide antihypertensive diuretics, loop diuretics, bronchodilators, drugs for gout and hyperuricemia, antimetabolites, and anti-tumor drugs) that could potentially impact serum UA concentration levels were excluded. Consequently, the analysis included a total of 1,738 Japanese individuals (799 men and 939 women) aged between 40 and 79 years.

### Assessment of mushroom consumption

In NHANES, dietary data were collected using up to two 24-h dietary recall interviews in which respondents reported all foods and beverages consumed during the preceding 24-h. Day 1 dietary recall data were collected in-person at the MEC by trained interviewers. Day 2 interviews were administered by telephone 3 to 10 days after MEC interviews. The 24-h dietary recalls were collected using the computerized method of the U.S. Department of Agriculture (USDA) Automated Multiple-Pass Method to account for day-to-day variation [22].

The U.S. Department of Agriculture Food and Nutrient Databases for Dietary Studies was used to determine the nutrient content of foods. Mushroom consumption was identified using USDA food codes that were mostly mushrooms or mushrooms alone, for example, egg omelet or scramble egg, with sausage and mushrooms, steak with mushrooms served on the side or dish that was mainly mushrooms, such as stuffed mushrooms sautéed mushrooms, creamed mushrooms. Since mushrooms are also incorporated into mixed dishes, the present analysis separated out mushrooms in mixed dishes using the U.S. Environmental Protection Agency-USDA Food Commodity Intake Database commodity codes [27]. Detailed information regarding the Food Commodity Intake Database and the process is described elsewhere [28–31].

In NILS-LSA, dietary assessments were conducted using 3-day dietary records (3DRs). These records required participants to document their dietary intake over a period of three consecutive days, encompassing two weekdays and one weekend day. The majority of participants completed these records at home and returned them within 1 month. During the assessment, food items were individually weighed using a 1-kg kitchen scales (Sekisui Jushi, Tokyo, Japan) before being cooked or portion sizes estimated. Additionally, participants used a disposable camera (27 shots; Fuji Film, Tokyo, Japan) to capture images of their meals before and after eating. Dietitians used these photos to complete missing data, and when necessary, they telephoned participants to clarify discrepancies or obtain further information, and calculated the average daily consumption of each food for each participant. Averages for 3-day nutrient and energy intakes were calculated according to the Standard Tables of Foods Composition 2010 in Japan and other sources [32].

### Ascertainment of hyperuricemia

In the continuous NHANES 2007–2018, serum UA was measured on a Beckman Synchron LX20 using an automated colorimetric method, which had been validated against a uricase assay [33]. Based on a previous study, hyperuricemia was defined in this study as serum UA levels > 420 μmol/L in men and > 350 μmol/L in women [21].

In NILS-LSA, venous blood was sampled using tubes containing ethylenediaminetetraacetic acid (disodium salt, 50 mM) in the morning immediately after fasting for ≥ 12 h. Serum UA (mg/dL) were measured by enzymatic method (Uricase-Peroxidase reaction) using the automatic analysis device (JCA-BM8000 Series; JEOL, Ltd.) [34]. The lower detection limit for UA was 0.2 mg/dL. Based on the Japanese guideline for the management of hyperuricemia and gout: second edition [35] and previous studies [36–38], hyperuricemia was defined as a serum UA level of > 7.0 mg/dL in men and ≥ 6.0 mg/dL in women. In order to facilitate comparison with NHANES, serum UA values were multiplied by 59.48 [39] to convert the units from mg/dL to μmol/L. That is, hyperuricemia was defined as > 416.4 μmol/L for men and ≥ 356.9 μmol/L for women.

### Assessment of covariates

Based on the previous literature, the following covariates were included in our analysis of NHANES: age (years), sex (men/women), ethnicity-race (Mexican American, other Hispanic, Non-Hispanic White as the referent group, other race-multi-racial), total physical activity (assessed according to metabolic equivalent of task (MET) scores (METs-h/day)), body mass index (BMI; kg/m^2^), education (less than high school, high school, above high school), family poverty income ratio (PIR; ≤ 1.30, > 1.30), smoking status (never, current smoker), alcohol intake (g/day), total energy intake (kcal/day), history of hypertension history of diabetes (yes or no for each), and consumption of fish and shellfish (oz/day), meat (oz/day), vegetables (svg/day), and fruit (svg/day). The family PIR is the ratio of family income to the poverty threshold. It is calculated by dividing the family income by poverty guidelines set forth by the U.S. Department of Health and Human Services, specific to family size, year, and state. BMI calculated as weight in kilograms divided by height in meters squared. Total physical activity was self-reported [40] and was measured using the Global Physical Activity Questionnaire [41]. The eGFR (mL/min/1.73m^2^) was calculated based on sex-age-specified serum creatinine (scr; mg/dL) (for men, if scr ≤ 0.9, eGFR = 142 × (scr/0.9)^-0.302^ × 0.9938^age^, and if scr > 0.9, eGFR = 142 × (scr/0.9)^-1.200^ × 0.9938^age^; for women, if scr ≤ 0.7, eGFR = 142 × (scr/0.7)^-0.241^ × 0.9938^age^ × 1.012, and if scr > 0.7, eGFR = 142 × (scr/0.7)^-1.200^ × 0.9938^age^ × 1.012.) [42]. Hemoglobin A1c (HbA1c) levels in NHANES were measured during the MEC by boronate affinity high-performance liquid chromatography (HPLC), which could differentiate between glycated and non-glycated forms of A1c as described in the NHANES Laboratory Procedure Manual, Glycohemoglobin [43]. Systolic and diastolic blood pressure was measured by trained certified blood pressure examiners using Mercury Sphygmomanometer with the participant in a seated position following 5 min of quiet rest. The average of 2nd and 3rd blood pressure was used for the present study [44, 45].

In the NILS-LSA, in each wave, data on age, disease history (kidney disease, gout, stroke, hypertension, heart disease, dyslipidemia, diabetes, cancer; yes or no for each), smoking status (never, former, current), and education level (years; grouped into three categories: less than high school (≤ 9 years), high school (10–12 years), and above high school (≥ 13 years)) were collected using self-reported questionnaires. Height (meters) and weight (kilograms) were measured using digital scales, and BMI (kg/m^2^) was calculated as weight divided by height squared. The 24-h total physical activity (METs-h/day) was METs-h/dayobtained from participant interviews conducted by trained interviewers using a semiquantitative assessment tool [46]. Food consumption (g/day) and alcohol intake (g/day) was measured by 3DRs, and medication data were recorded according to the participant's prescription drug pick-up booklet. Serum concentration of creatinine was tested and eGFR (mL/min/1.73m^2^) was calculated using same equations with NHANES. HbA1c levels were measured using a latex aggregation immunoassay (SRL, Tokyo, Japan) with venous blood samples collected in the early morning following a minimum 12-h fast. The HbA1c values were calculated as National Glycohemoglobin Standardization Program equivalent values using the following formula: HbA1c (%) = 1.02 × HbA1c (Japan Diabetes Society) (%) + 0.25% [47]. Systolic and diastolic blood pressure was measured with an automatic blood pressure manometer (BP-204RV, Colin, Inc.) while subjects were in a seated position, following at least 5 min of rest.

### Statistical analysis

In the NHANES, mushroom consumption (g/day) were divided into three groups: the non-consumer group (0 g/day, *n* = 5,516), the middle group (median intake = 4.4 g/day, *n* = 133), and the highest group (median intake = 19.6 g/day,* n* = 129). The non-consumer group was considered the referent group.

Consistent with the NCHS guidelines for analyzing the NHANES data, all analyses were conducted using appropriate sampling weights, clustering, and stratification to account for the complex sampling design [22]. Univariable analyses were performed to assess the statistical differences in weighted percentages for categorical variables using the Rao-Scott χ2 test and weighted means for continuous variables using analysis of variance to describe the characteristics of the study participants. We performed multivariable logistic regression models stratified by gender using the SAS surveylogistic procedure to examine the association of mushroom consumption with hyperuricemia. Model 1 was adjusted for baseline information on age (years; continuous), ethnicity-race (Mexican American, other Hispanic, Non-Hispanic White, Non-Hispanic Black, other race), education (less than high school, high school, above high school), family PIR (≤ 1.30, > 1.30), BMI (kg/m^2^; < 24.9, 25.0–29.9, ≥ 30.0), disease history (hypertension, diabetes; yes or no for each), eGFR (mL/min/1.73m^2^; continuous). Model 2 was adjusted for the covariates specified in Model 1 as well as baseline information on smoking status (never, former, current, alcohol drinking (g/day, and total physical activity (METs-h/day; continuous). Parsimonious Model 3 was adjusted for Model 2 plus baseline information on consumption (g/day; continuous) of fish and shellfish, meat, vegetables, and fruit, and energy intake (kcal/d; continuous). The multivariable logistic regression results were presented as adjusted proportional odds ratio (pOR) and the 95% confidence intervals (95% CIs).

In the NILS-LSA, mushroom consumption (g/day) were divided into three groups: the non-consumer group (0 g/day;* n* = 327), the middle group (< sex-specific median value (13.3 g/day for both gender); median intake = 6.7 g/day, *n* = 699), and the highest group (≥ sex-specific median value; median intake = 22.9 g/day, *n* = 718). The non-consumer group was considered the referent group.

To investigate associations between mushroom consumption and hyperuricemia, multivariable-adjusted generalized estimation equation were used to calculate OR and 95% CI by gender. Model 1 was adjusted for baseline information on age (years; continuous), BMI (kg/m^2^; < 18.5, 18.5– < 25, ≥ 25), disease history (hypertension, dyslipidemia, diabetes; yes or no for each), eGFR (mL/min/1.73m^2^; continuous), serum UA concentration level (μmol/L), education (less than high school, high school, above high school), and follow-up time (days; continuous). Model 2 was adjusted for the covariates specified in Model 1 as well as baseline information on smoking status (current smoker, others), alcohol drinking (current drinker, others), and total physical activity (METs-h/day; continuous). Model 3 was adjusted for Model 2 plus baseline information on consumption (g/day; continuous) of fish and shellfish, meat, vegetables, and fruit, and energy intake (kcal/d; continuous).

In the above analysis, we observed a potential preventive effect of mushroom consumption on hyperuricemia among men in NILS-LSA. Considering previous evidence indicating a higher risk of hyperuricemia in middle-aged population [48], we performed age-stratified analyses (for the middle-aged subgroup < 65 years and the older subgroup ≥ 65 years) among men in NILS-LSA.

Statistical analysis was performed using SAS software (version 9.4-SAS institute) and R Statistical Software (version 4.3.0; R Foundation for Statistical Computing, Vienna, Austria). Two-sided exact *P-*values of < 0.05 were considered the threshold for statistical significance.

## Results

### Characteristics of participants

A total of 5,778 NHANES participants (mean (SD) age: 53.2 (9.6) years) were included in the present analysis. Mushrooms were consumed by 5.7% (95% CI: 4.7%, 6.7%) of NHANES participants. From the descriptive analysis, more than half (51.0%) of the study participant were women. About 74.2% were non-Hispanic White, and 66.8% had more than a high school degree. Participants with higher mushroom intake were more likely to be Non-Hispanic White, and had higher fruit and vegetable intake than those with those with no mushroom intake (Table 1). Moreover, there were statistically significant differences between mushroom consumption groups for family PIR, smoking status, and total physical activity, HbA1c, and systolic blood pressure (Table 1).

**Table 1:** Characteristics of the study participants aged ≥ 40 years in NHANES 2007–2018 (*n* = 5,778)^a^

	Groups of mushroom consumption			*P*-value^b^
	Non-consumer	Middle	Highest	
No. Participants	5,516	133	129	
Mushroom consumption (g/day); mean (SD)	0 -	4.5 (2.8)	24.7 (20.3)	< 0.001
Serum UA (μmol/L); mean (SD)	309.7 (75.3)	299.3 (78.9)	315.2 (76.8)	0.690
Age (years); mean (SD)	53.2 (9.7)	52.6 (9.2)	53.1 (8.9)	0.822
Men; %	49.8	36.9	41.0	0.104
BMI (kg/m^2^); mean (SD)	27.9 (5.8)	28.1 (6.4)	28.0 (7.2)	0.988
Estimated glomerular filtration rate (eGFR; mL/min/1.73m^2^; continuous); mean (SD)	93.9 (14.0)	96.2 (12.6)	95.5 (12.8)	0.127
HbA1c (%); mean (SD)	5.6 (0.7)	5.5 (0.4)	5.5 (0.4)	0.037
Systolic blood pressure (mm Hg); mean (SD)	122.1 (16.1)	118.4 (12.8)	117.8 (11.7)	0.001
Diastolic blood pressure (mm Hg); mean (SD)	73.0 (10.9)	72.7 (10.1)	71.1 (8.8)	0.152
Non-Hispanic White; %	73.6	84.6	83.5	0.004
Family PIR ≤ 1.30; %	15.1	8.4	7.1	0.015
Medical history (yes); %				
Hypertension	11.8	6.4	8.8	0.303
Diabetes mellitus	4.6	2.5	1.4	0.218
Current smoker; %	18.9	8.2	14.7	0.061
Alcohol intake (g/day); mean (SD)	10.6 (22.2)	11.6 (22.4)	15.7 (20.9)	0.194
Total physical activity (METs-h/day); mean (SD)	10.5 (14.0)	9.0 (11.1)	8.1 (10.7)	0.075
Education above high school; %	66.1	73.3	82.6	0.051
Energy intake (kcal/day); mean (SD)	2,085.6 (706.1)	2,112.2 (623.7)	2,145.8 (680.9)	0.784
Food consumption				
Fish and shellfish (oz/day); mean (SD)	0.7 (1.7)	0.8 (1.7)	0.8 (1.3)	0.853
Meat (oz/day); mean (SD)	2.6 (2.4)	2.2 (2.3)	2.3 (2.5)	0.258
Vegetables (svg/day); mean (SD)	0.6 (0.6)	0.6 (0.6)	0.9 (0.6)	< 0.001
Fruit (svg/day); mean (SD)	1.1 (1.2)	1.0 (1.2)	1.6 (1.6)	0.078

^a^Weighted mean (standard deviation) for continuous variable, and weighted proportion for categorical variable

^b^For continuous variables, the Analysis of Variance (ANOVA) was used; for categorical variables, the Rao Scott χ^2^ test was used

A total of 1,738 NILS-LSA participants (mean (SD) age of 53.5 (11.2) years) were included. Mushrooms were consumed by 81.2% of NILS-LSA participants. Compared to non-consumers, participants with higher mushroom consumption also consumed more fish, shellfish, and vegetables (Table 2).

**Table 2:** Baseline characteristics of participants in NILS-LSA (*n* = 1,738)

	Groups of mushroom consumption			*P*-value^a^
	Non-consumer	Middle	Highest	
No. of participants	326	696	716	
Mushroom consumption (g/day); mean (SD)	0 -	6.7 (3.3)	26.7 (13.0)	< 0.001
Serum UA (μmol/L); mean (SD)	283.2 (64.2)	281.1 (64.0)	279.2 (64.4)	0.645
Age (years); mean (SD)	53.7 (11.7)	53.1 (11.4)	53.9 (10.9)	0.406
Men; %	43.6	46.8	46.2	0.608
BMI (kg/m^2^); mean (SD)	22.6 (3.0)	22.5 (2.9)	22.6 (2.8)	0.907
Estimated glomerular filtration rate (eGFR; mL/min/1.73m^2^; continuous); mean (SD)	97.8 (14.1)	98.1 (14.7)	97.6 (14.2)	0.840
HbA1c (%); mean (SD)	5.5 (0.9)	5.5 (0.6)	5.5 (0.6)	0.688
Systolic blood pressure (mm Hg); mean (SD)	119.5 (18.1)	119.9 (19.1)	119.2 (17.9)	0.785
Diastolic blood pressure (mm Hg); mean (SD)	73.6 (10.8)	73.7 (11.1)	73.6 (10.9)	0.957
Medical history (yes); %				
Hypertension	17.5	14.9	14.1	0.365
Dyslipidemia	10.7	11.6	12.7	0.633
Diabetes mellitus	4.9	3.6	6.4	0.051
Current smoker; %	20.9	22.8	18.9	0.182
Alcohol intake (g/day); mean (SD)	10.2 (20.2)	10.1 (16.0)	9.3 (14.5)	0.539
Total physical activity (METs-h/day); mean (SD)	36.1 (4.2)	36.1 (4.1)	36.0 (4.1)	0.888
Education above high school; %	35.6	37.2	37.6	0.665
Energy intake (kcal/day); mean (SD)	2104.6 (430.4)	2138.5 (410.8)	2149.0 (410.8)	0.272
Food consumption (g/day); mean (SD)				
Fish and shellfish	85.7 (49.8)	93.2 (51.6)	101.1 (52.0)	< 0.001
Meat	35.0 (28.0)	36.4 (28.1)	36.5 (29.6)	0.729
Vegetables	251.2 (118.9)	255.2 (101.7)	292.2 (115.2)	< 0.001
Fruit	135.0 (134.2)	126.9 (109.7)	140.1 (114.2)	0.102

^a^For continuous variables, the Analysis of Variance (ANOVA) was used; for categorical variables, the χ^2^ test was used

### Cross-sectional relationship among men and women in the NHANES

In NHANES, no associations were observed for U.S. men and women after adjusting for potential confounders. The corresponding proportional odds ratio (pOR) (95% CI) for 0 g/day, mean intake (4.3 g/day), and mean intake (27.2) among men were 1.00 (Ref.), 0.81 (0.23, 2.89), 1.17 (0.43, 3.24), respectively (*P*-trend = 0.827) and for 0 g/day, mean intake (4.7 g/day), and mean intake (23.0 g/day) among women were 1.00 (Ref.), 0.71 (0.20, 2.47), 1.07 (0.48, 2.37), respectively (*P*-trend = 0.982) (Table 3).

**Table 3:** ORs and corresponding 95% CIs for hyperuricemia by mushroom consumption (NHANES; 2007–2018; *n* = 5,778)^a^

	Groups of mushroom consumption								*P*-trend
	Non-consumer		Middle			Highest			
	pOR	95% CI	pOR	95% CI		pOR	95% CI		
Men (*n* = 2,934)									
Mushroom consumption (g/day); mean (SD)	0 (-)		4.3 (2.9)			27.2 (26.3)			
No. of participants	2833		51			50			
Prevalence of hyperuricemia; %	14.8		11.1			15.8			
Model 1^b^	1.00	Ref.	0.74	0.22	2.52	1.22	0.41	3.62	0.793
Model 2^c^	1.00	Ref.	0.79	0.23	2.69	1.16	0.38	3.54	0.871
Model 3^d^	1.00	Ref.	0.81	0.23	2.89	1.17	0.43	3.24	0.827
Women (*n* = 2,844)									
Mushroom consumption (g/day); mean (SD)	0 (-)		4.7 (2.8)			23.0 (14.8)			
No. of participants	2683		82			79			
Prevalence of hyperuricemia; %	10.2		5.9			11.8			
Model 1^b^	1.00	Ref.	0.69	0.21	2.28	1.25	0.58	2.70	0.686
Model 2^c^	1.00	Ref.	0.67	0.20	2.24	1.21	0.55	2.63	0.767
Model 3^d^	1.00	Ref.	0.71	0.20	2.47	1.07	0.48	2.37	0.982

^a^Analysis by SAS survey procedure (surveylogistic)

^b^Adjusted for age (years; continuous), ethnicity-race (Mexican American, other Hispanic, White Non-Hispanic, Black Non-Hispanic, other race), education (less than high school, high school, above high school), family PIR (≤ 1.30, > 1.30), BMI (kg/m^2^; < 24.9, 25.0–29.9, ≥ 30.0), disease history (hypertension, diabetes; yes or no for each), eGFR (mL/min/1.73m^2^; continuous)

^c^Adjusted for Model 1 + smoking status (never, former, current), alcohol drinking (g/day; continuous), and total physical activity (METs-h/day; continuous)

^d^Adjusted for Model 2 + consumption (continuous) of fish and shellfish (oz/day), meat (oz/day), vegetables (svg/day), and fruit (svg/day), and energy intake (kcal/d; continuous)

### Prospective relationship among men and women in the NILS-LSA

The number and prevalence of hyperuricemia during the follow-up period, categorized by mushroom consumption in NILS-LSA according to gender, are presented in Supplementary Table 1. In the NILS-LSA, although no association between mushroom consumption and the incident hyperuricemia was observed in either men or women (Table 4), among men aged under 65 years, compared to non-consumers, a higher mushroom intake was associated with a lower risk of hyperuricemia. Multivariable-adjusted OR (95% CI) for non-consumers, participants with middle, and participants with the highest consumption of mushroom were 1.00 (Ref.), 0.77 (0.44, 1.36), 0.55 (0.31, 0.99), respectively (*P*-trend = 0.036). However, due to limitations imposed by the sample size and the small number of participants who developed incident hyperuricemia during the follow-up period (Supplementary Table 2), we were unable to observe any associations in men aged 65 years and above (Table 5).

**Table 4:** ORs and corresponding 95% CIs for incident hyperuricemia by mushroom consumption (NILS-LSA; *n* = 1,738)^a^

	Groups of mushroom consumption								*P*-trend
	Non-consumer		Middle			Highest			
	OR	95% CI	OR	95% CI		OR	95% CI		
Men (*n* = 799)									
Mushroom consumption (g/day); mean (SD)	0 (-)		6.8 (3.3)			26.9 (12.9)			
No. of participants	142		326			331			
Model 1^b^	1.00	Ref.	0.81	0.48	1.38	0.67	0.39	1.13	0.120
Model 2^c^	1.00	Ref.	0.79	0.47	1.32	0.67	0.40	1.13	0.135
Model 3^d^	1.00	Ref.	0.74	0.43	1.27	0.65	0.38	1.12	0.129
Women (*n* = 939)									
Mushroom consumption (g/day); mean (SD)	0 (-)		6.6 (3.2)			26.5 (13.1)			
No. of participants	184		370			385			
Model 1^b^	1.00	Ref.	0.94	0.51	1.74	0.86	0.46	1.60	0.620
Model 2^c^	1.00	Ref.	0.99	0.53	1.86	0.89	0.48	1.66	0.684
Model 3^d^	1.00	Ref.	1.03	0.55	1.91	0.88	0.47	1.63	0.613

^a^Analysis by Generalized estimating equation

^b^Adjusted for baseline information on age (years; continuous), BMI (kg/m^2^; < 18.5, 18.5– < 25, ≥ 25), disease history (hypertension, dyslipidemia, diabetes; yes or no for each), eGFR (mL/min/1.73m^2^; continuous), serum UA concentration level (μmol/L), education (less than high school, high school, above high school), and follow-up time (days; continuous)

^c^Adjusted for Model 1 + baseline information on smoking status (current smoker, others), alcohol drinking (current drinker, others), and total physical activity (METs-h/day; continuous)

^d^Adjusted for Model 2 + baseline information on consumption (g/day; continuous) of fish and shellfish, meat, vegetables, and fruit, and energy intake (kcal/d; continuous)

**Table 5:** ORs and corresponding 95% CIs for incident hyperuricemia by mushroom consumption in men by age (NILS-LSA; *n* = 799)

	Groups of mushroom consumption								*P*-trend
	Non-consumer		Middle			Highest			
	OR	95% CI	OR	95% CI		OR	95% CI		
< 65 years (*n* = 633)									
Mushroom consumption (g/day); mean (SD)	0 (-)		6.8 (3.4)			26.8 (12.9)			
No. of participants	111		253			269			
Model 1^b^	1.00	Ref.	0.85	0.50	1.45	0.57	0.33	0.99	0.034
Model 2^c^	1.00	Ref.	0.81	0.48	1.38	0.58	0.33	1.01	0.042
Model 3^d^	1.00	Ref.	0.77	0.44	1.36	0.55	0.31	0.99	0.036
≥ 65 years (*n* = 166)									
Mushroom consumption (g/day); mean (SD)	0 (-)		6.8 (3.0)			27.3 (13.1)			
No. of participants^e^	31		73			62			

^a^Analysis by Generalized estimating equation

^b^Adjusted for baseline information on age (years; continuous), BMI (kg/m^2^; < 18.5, 18.5– < 25, ≥ 25), disease history (hypertension, dyslipidemia, diabetes; yes or no for each), eGFR (mL/min/1.73m^2^; continuous), serum UA concentration level (μmol/L), education (less than high school, high school, above high school), and follow-up time (days; continuous)

^c^Adjusted for Model 1 + baseline information on smoking status (current smoker, others), alcohol drinking (current drinker, others), and total physical activity (METs-h/day; continuous)

^d^Adjusted for Model 2 + baseline information on consumption (g/day; continuous) of fish and shellfish, meat, vegetables, and fruit, and energy intake (kcal/d; continuous)

^e^The number of participants in each subgroup and the count of those who developed hyperuricemia during the follow-up period was too small for the model to converge. As a result, the OR could not be calculated

## Discussion

In this large collaborative study of U.S. and Japan, mushroom consumption was associated with a lower risk of incident hyperuricemia in middle-aged Japanese men. In the NHANES study, no such association was observed for U.S. men and women. This discrepancy may be attributed to the fact that mushrooms were consumed by 5.7% of NHANES participants and 81.2% of NILS-LSA participants, indicating significant differences in the proportion of individuals habitually consuming mushrooms between the two study populations. Furthermore, the variations in the results by age in Japanese men suggest that for middle-aged men, mushroom consumption may be an effective way to prevent hyperuricemia. Conversely, for older men, alternative and more effective methods of hyperuricemia prevention might be required.

To our knowledge, so far, only one epidemiological study based on a community population has explored the association between mushroom consumption and the incidence of hyperuricemia. This study, conducted in China, indicated that consuming mushrooms is associated with a reduced risk of developing hyperuricemia [21], which is consistent with our findings in middle-aged Japanese men. We observed differences in the association between mushroom consumption and the incident hyperuricemia among middle-aged and older Japanese men. In addition to being influenced by the sample size, these differences may also be attributed to variations in the prevalence of hyperuricemia among different age groups. Study conducted among the Japanese population during the same period as NILS-LSA's data collection has shown that the prevalence of hyperuricemia is significantly higher in individuals under 60 years of age compared to those aged 60 and above [49]. A prior study conducted in the Chinese population has also indicated a positive association between the risk of hyperuricemia and younger age [48]. This study shows that the higher prevalence of hyperuricemia among the younger population might be attributed to unhealthy dietary habits and increased participation in social activities involving heavy alcohol consumption.

While the incidence of hyperuricemia was not measured in the U.S. NHANES, we observed no relationship with mushroom consumption in the cross-sectional analysis. The lack of a significant association between mushroom consumption and hyperuricemia in the U.S. population could be attributed to two factors. First, mushroom consumption is relatively low in the U.S. compared to other Asian countries such as China, Japan, and Korea, where mushrooms are widely consumed as a staple food and medicine [29]. According to a previous study, the per capita consumption of fresh mushrooms in the U.S. was slightly less than 3 pounds (about 1.4 kg) per year [15], while the average annual consumption expenditure of mushrooms reached 13 kg in Japan [50]. In this study, although the average daily consumption of mushrooms did not differ significantly between American and Japanese subjects, the proportion of mushrooms consumed by American participants was substantially lower than that of Japanese participants. Thus, in the long term, it may be that the average annual consumption of mushrooms in the U.S. is too low to have a strong biological effect on uric acid levels. Secondly, the preventive effect of high mushroom intake on hyperuricemia shown in studies conducted in China and Japan may be attributed to the fact that the types of mushrooms commonly consumed in these countries differ from those in the U.S. In Japan, shiitake, oyster, maitake, and king oyster mushrooms are often consumed while white button, cremini, and portabellas mushrooms dominate the U.S. mushroom market [18]. Since no studies to date have quantified the biochemical components (e.g., xanthine inhibitors) of specific species of mushrooms that inhibit elevated uric acid, we cannot determine the preventive mechanism of mushrooms against hyperuricemia yet.

Regarding the gender differences reflected in the association between mushroom consumption and hyperuricemia, we have two possible speculations. The first speculation is based on the evident gender differences in the physiological regulation of uric acid balance. Female sex hormones, specifically estrogen, may play a role in the regulation of expression or activity of UA transporters. Estrogen can reduce the reabsorption of UA in the kidneys, thereby promoting its excretion through urine [51]. The second speculation is that the gender difference is a result of different lifestyles and dietary habits between women and men. Although we adjusted for potential influencing factors in the analysis, among the participants in the NILS-LSA, the proportions of women with diabetes and women who are current smokers were significantly lower than that of men. Moreover, the daily consumption of fish and shellfish, meat, as well as alcohol and energy intake, were much lower in women than in men. On the contrary, women consumed more vegetables and fruits compared to men. Therefore, consuming mushrooms may potentially provide greater benefits in preventing hyperuricemia for men compared to women.

One possible mechanism for the potential preventive effect of consuming mushrooms against hyperuricemia could be attributed to the presence of xanthine inhibitors in mushrooms, which can lower serum uric acid levels [20]. Intriguingly, a study has indicated that the purine content in fungi, especially after drying, is very high [52]. Conversely, other researchers have suggested that the purine content in mushrooms (excluding *hiratake* and dried *shiitake*) is quite low [53]. Evidence has also indicated that consuming purine-rich fungi did not affect the occurrence of hyperuricemia in Chinese population [54].

To the best of our knowledge, this is the first collaborative study to use a nationally representative sample of the U.S. and the Japanese middle-aged and older community-dwelling population. However, our study does have some limitations that need to be addressed. First, mushroom consumption in this study was estimated using one- or two-day dietary recalls and 3-day dietary records, which may not have adequately captured the actual usual consumption over time. Second, information about different mushroom species consumed by participants was not available. Third, even though we controlled for major potential confounders, residual confounding is possible in observational studies. Lastly, the cross-sectional design of NHANES data precludes establishing a clear temporal relationship between mushroom intake and hyperuricemia.

## Conclusions

In this large collaborative study including NHANES data from the U.S. and NILS-LSA data from Japan, we found that mushroom consumption was associated with a lower risk of incident hyperuricemia in middle-aged Japanese men but not for the U.S. population. Future large scale epidemiological research with repeated dietary data measurements and hyperuricemia diagnostic data is needed to replicate our findings and further confirm the beneficial effects of mushroom consumption in preventing hyperuricemia.

### Supplementary Information

**Additional file 1:Supplementary Table 1.** The number and prevalence of hyperuricemia during the follow-up period, categorized by mushroom consumption and stratified by gender (NILS-LSA; *n* = 1,738). **Supplementary Table 2.** The number and prevalence of hyperuricemia during the follow-up period, categorized by mushroom consumption and stratified by age in men (NILS-LSA; *n* = 799).

## Data Availability

The NHANES datasets used and/or analyzed during the current study are available at https://wwwn.cdc.gov/nchs/nhanes/. The data of NILS-LSA analyzed in the current study are not publicly available for privacy reasons but are available from the corresponding author upon reasonable request.

## Abbreviations

BMI: Body mass index
95% CIs: 95% Confidence intervals
3DRs: 3-Day dietary records
eGFR: Estimated Glomerular Filtration Rate
g: Grams
HbA1c: Hemoglobin A1c
MEC: Mobile examination center
MET: Metabolic equivalent of task
NHANES: National health and nutrition examination survey
NILS-LSA: National institute for longevity sciences-longitudinal study of aging
NCHS: National center for health statistics
ORs: Odds ratios
PIR: Poverty income ratio
pOR: Proportional odds ratio
scr: Serum creatinine
U.S.: United States
USDA: U.S. Department of agriculture
