# Supplementary material for: Mushroom consumption and hyperuricemia: results from the National Institute for Longevity Sciences-Longitudinal Study of Aging and the National Health and Nutrition Examination Survey (2007-2018)
Source: Nutr J. 2023 Nov 22;22:62. doi: 10.1186/s12937-023-00887-0 (PMC10664361; doi:10.1186/s12937-023-00887-0)
Supplement: Supplementary file 1 — Additional file 1:Supplementary Table 1. The number and prevalence of hyperuricemia during the follow-up period, categorized by mushroom consumption and stratified by gender (NILS-LSA; n = 1,738). Supplementary Table 2. The number and prevalence of hyperuricemia during the follow-up period, categorized by mushroom consumption and stratified by age in men (NILS-LSA; n = 799). [file 12937_2023_887_MOESM1_ESM.docx]

| **Supplementary Table 1**. The number and prevalence of hyperuricemia during the follow-up period, categorized by mushroom consumption and stratified by gender (NILS-LSA; n = 1,738). | | | | | | | | | |
| --- | --- | --- | --- | --- | --- | --- | --- | --- | --- |
| Follow-up visits | | Groups of mushroom consumption | | | | | | | |
|  |  | Non-consumer | |  | Middle | |  | Highest | |
|  |  | No. of participants | No. (%) of hyperuricemia |  | No. of participants | No. (%) of hyperuricemia |  | No. of participants | No. (%) of hyperuricemia |
| Men (n = 799) | |  |  |  |  |  |  |  |  |
|  | 0 (Baseline) | 142 | 0 (-) |  | 326 | 0 (-) |  | 331 | 0 (-) |
|  | 1 | 131 | 12 (9.2%) |  | 313 | 24 (7.7%) |  | 309 | 20 (6.5%) |
|  | 2 | 132 | 14 (10.6%) |  | 302 | 32 (10.6%) |  | 309 | 24 (7.8%) |
|  | 3 | 116 | 9 (7.8%) |  | 269 | 27 (10.0%) |  | 271 | 24 (8.9%) |
|  | 4 | 98 | 13 (13.3%) |  | 225 | 21 (9.3%) |  | 240 | 17 (7.1%) |
|  | 5 | 80 | 9 (11.3%) |  | 170 | 18 (10.6%) |  | 202 | 14 (6.9%) |
|  | 6 | 56 | 9 (16.1%) |  | 132 | 9 (6.8%) |  | 163 | 10 (6.1%) |
| Women (n = 939) | |  |  |  |  |  |  |  |  |
|  | 0 (Baseline) | 184 | 0 (-) |  | 370 | 0 (-) |  | 385 | 0 (-) |
|  | 1 | 175 | 7 (4.0%) |  | 342 | 7 (2.0%) |  | 357 | 6 (1.7%) |
|  | 2 | 169 | 8 (4.7%) |  | 349 | 10 (2.9%) |  | 353 | 14 (4.0%) |
|  | 3 | 147 | 8 (5.4%) |  | 302 | 11 (3.6%) |  | 321 | 10 (3.1%) |
|  | 4 | 117 | 8 (6.8%) |  | 251 | 11 (4.4%) |  | 275 | 12 (4.4%) |
|  | 5 | 87 | 4 (4.6%) |  | 195 | 10 (5.1%) |  | 220 | 10 (4.5%) |
|  | 6 | 72 | 4 (5.6%) |  | 163 | 14 (8.6%) |  | 158 | 9 (5.7%) |

| **Supplementary Table 2**. The number and prevalence of hyperuricemia during the follow-up period, categorized by mushroom consumption and stratified by age in men (NILS-LSA; n = 799). | | | | | | | | | |
| --- | --- | --- | --- | --- | --- | --- | --- | --- | --- |
| Follow-up visits | | Groups of mushroom consumption | | | | | | | |
|  |  | Non-consumer | |  | Middle | |  | Highest | |
|  |  | No. of participants | No. (%) of hyperuricemia |  | No. of participants | No. (%) of hyperuricemia |  | No. of participants | No. (%) of hyperuricemia |
| < 65 years (n = 633) | |  |  |  |  |  |  |  |  |
|  | 0 (Baseline) | 111 | 0 (-) |  | 253 | 0 (-) |  | 269 | 0 (-) |
|  | 1 | 102 | 10 (9.8%) |  | 243 | 20 (8.2%) |  | 248 | 16 (6.5%) |
|  | 2 | 101 | 12 (11.9%) |  | 233 | 24 (10.3%) |  | 250 | 20 (8.0%) |
|  | 3 | 92 | 8 (8.7%) |  | 219 | 22 (10.0%) |  | 222 | 18 (8.1%) |
|  | 4 | 82 | 12 (14.6%) |  | 193 | 19 (9.8%) |  | 202 | 14 (6.9%) |
|  | 5 | 69 | 8 (11.6%) |  | 149 | 16 (10.7%) |  | 174 | 10 (5.7%) |
|  | 6 | 51 | 9 (17.6%) |  | 119 | 9 (7.6%) |  | 141 | 7 (5.0%) |
| ≥ 65 years (n = 166) | |  |  |  |  |  |  |  |  |
|  | 0 (Baseline) | 31 | 0 (-) |  | 73 | 0 (-) |  | 62 | 0 (-) |
|  | 1 | 29 | 2 (6.9%) |  | 70 | 4 (5.7%) |  | 61 | 4 (6.6%) |
|  | 2 | 31 | 2 (6.5%) |  | 69 | 8 (11.6%) |  | 59 | 4 (6.8%) |
|  | 3 | 24 | 1 (4.2%) |  | 50 | 5 (10.0%) |  | 49 | 6 (12.2%) |
|  | 4 | 16 | 1 (6.3%) |  | 32 | 2 (6.3%) |  | 38 | 3 (7.9%) |
|  | 5 | 11 | 1 (9.1%) |  | 21 | 2 (9.5%) |  | 28 | 4 (14.3%) |
|  | 6 | 5 | 0 (-) |  | 13 | 0 (-) |  | 22 | 3 (13.6%) |
